# gganatogram: An R package for modular visualisation of anatograms and tissues based on ggplot2

**DOI:** 10.12688/f1000research.16409.2

**Published:** 2018-11-20

**Authors:** Jesper L.V. Maag

**Affiliations:** 1 Center for Epigenetics Research, Memorial Sloan Kettering Cancer Center, New York, New York, 10065, USA

**Keywords:** Anatograms, Anatomy, Tissues, Organs, ggplot2, R, Expression Atlas, Shiny

## Abstract

Displaying data onto anatomical structures is a convenient technique to quickly observe tissue related information. However, drawing tissues is a complex task that requires both expertise in anatomy and the arts. While web based applications exist for displaying gene expression on anatograms, other non-genetic disciplines lack similar tools. Moreover, web based tools often lack the modularity associated with packages in programming languages, such as R.

Here I present gganatogram, an R package used to plot modular species anatograms based on a combination of the graphical grammar of ggplot2 and the publicly available anatograms from the Expression Atlas. This combination allows for quick and easy, modular, and reproducible generation of anatograms. Using only one command and a data frame with tissue name, group, colour, and  value, this tool enables the user to visualise specific human and mouse tissues with desired colours, grouped by a variable, or displaying a desired value, such as gene-expression, pharmacokinetics, or bacterial load across selected tissues. gganatogram consists of 5 highly annotated organisms, male/female human/mouse, and a cell anatogram. It further consists of 24 other less annotated organisms from the animal and plant kingdom. I hope that this tool will be useful by the wider community in biological sciences. Community members are welcome to submit additional anatograms, which can be incorporated into the package.

A stable version gganatogram has been deposited to neuroconductor, and a development version can be found on github/jespermaag/gganatogram. An interactive shiny app of gganatogram can be found on https://jespermaag.shinyapps.io/gganatogram/, which allows for non-R users to create anatograms.

## Introduction

Efficiently displaying tissue information in multicellular organisms can be a laborious and time consuming process. Often researchers want to showcase differences in values, such as gene expression or pharmacokinetics between tissues in one organism, or between similar tissues in different groups.

**Figure 1.**
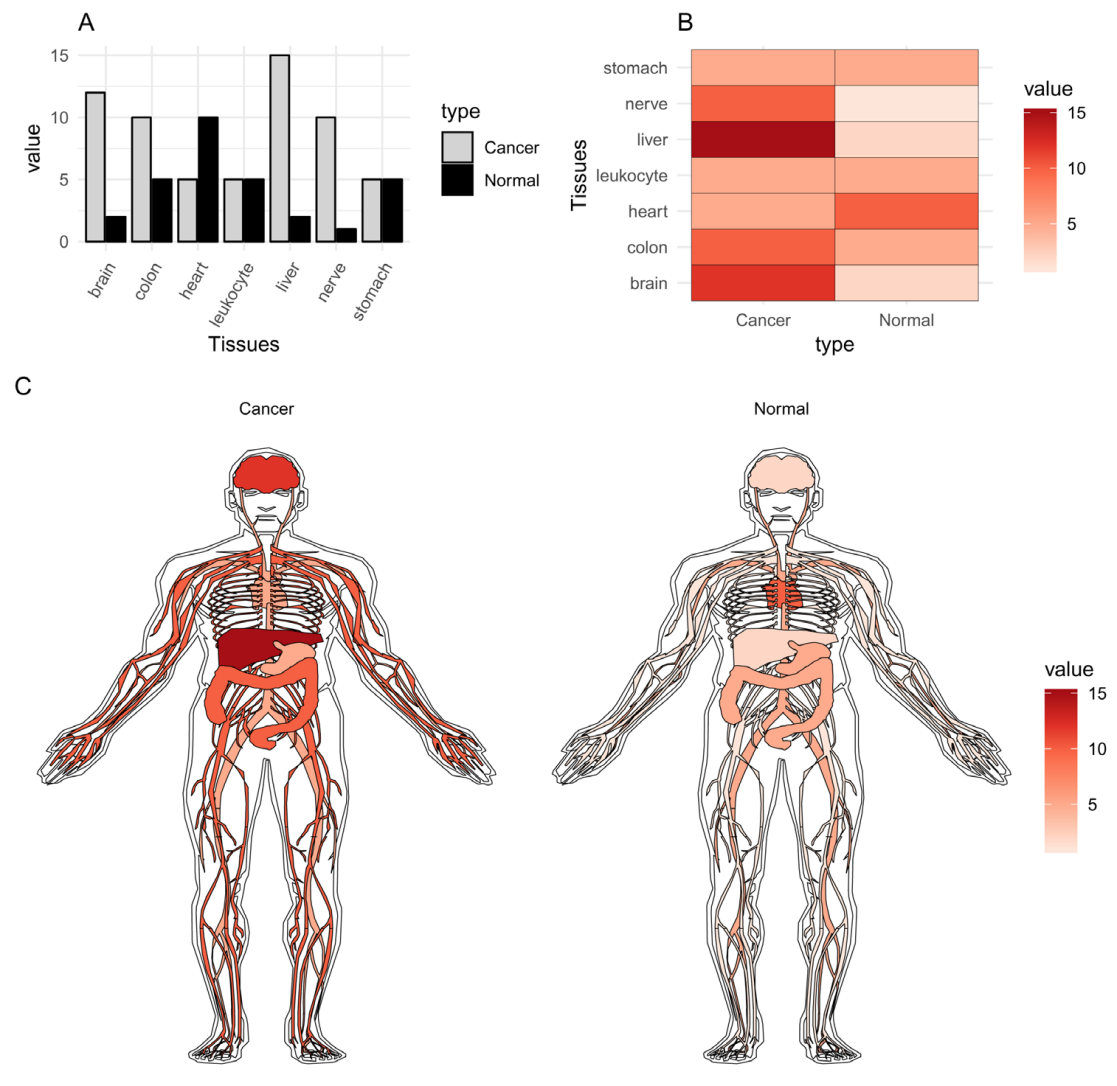
Comparison between barplot (top left), heatmap (top right), and anatogram (bottom) to display tissue values between groups. The values in the graphs are the same.

Whereas bar charts and heatmaps provide an informative view of the differences between groups, it can be difficult to immediately observe the biological significance ( Figure 1a–b). As compared to an anatogram, where it is easy to quickly spot the differences between tissues or groups, and immediately provide biological context to these observations ( Figure 1c). This also has the added benefit that the audience, whether reading a paper or attending a lecture, will have to spend less time and effort to grasp the results.

Several online tools to display gene expression in different tissues already exist ^1– 4^. Although these tools provide important information regarding gene expression in various tissues and organisms, other disciplines besides genetics are unable to utilise these applications due to the focus on genes. Moreover, these tools often only include a predefined set of experiments that can be visualised, leading to difficulties in presenting your own data. Other caveats with these tools are that it can be laborious to recreate the plot or automatically create plots from results.

Here I present gganatogram, an open source R package based on ggplot2 ^5^ utilising 28 publicly available anatograms from the Expression Atlas ^1, 2^, and a cellular anatogram from The Protein Atlas ^6^. With this package it is easy for any R user to quickly visualise anatograms with specified colours, groups, and values. Using the familiar grammar from ggplot2 ^5^, this program allows for modular anatograms to be generated.

## Methods

### Implementation

gganatogram is stored on neuroconductor ^7^, an open-source platform for rapid testing and dissemination of reproducible computational imaging software. A development version can be found on github/jespermaag/gganatogram, which allows for the community to post issues with the package, submit requests, or add anatograms by creating coordinate files.

source("https://neuroconductor.org/neurocLite.R")
neuro_install("gganatogram")

The development version can be installed from github:

devtools::install_github("jespermaag/gganatogram")

Briefly, to generate the main list objects that contain all tissue coordinates, I downloaded SVG files from the Expression Atlas ( SVG files present here ^2^. (and processed them using a custom python script (available from GitHub). The script scraped through the SVG files to extract the name, coordinates, and SVG transformations. These were then post-processed in R to create the rda files that make up the tissue coordinates. For the cell, the SVG was downloaded from The Protein Atlas ^6^. Here, I converted the relative coordinates in the SVG to absolute using Inkscape. I then processed the absolute coordinate SVG using python.

### Operation

gganatogram requires an installation of R *≥*3.0.0, ggplot2 ^5^ v.3.0.0 and ggpolypath ^8^ v.0.1.0. The program should be able to run on any computer with the system requirements for R. The online shiny version does not require an installation of R since it is run on the server side.

Plots can be generated using a basic data.frame containing organ name, colour, type, or value, with the specified column names below. Organs are plotted one at a time based on the order of the data.frame. The tissue of each consecutive row will be layered on top of the previous. The gganatogram package provides 29 such data.frames containing all tissues available to plot, one for each human and mouse, divided by sex, one cell, and 24 other organisms ( Table 1).

hgMale_key, hgFemale_key, mmMale_key, mmFemale_key, cell_key[["cell"]]

**Table 1.** Showing all the organisms in gganatogram, how to call their keys, and the number of features per organisms.

	Organism	Key	Number of features
1	Human male	hgMale_key	68
2	Human female	hgFemale_key	70
3	Mouse male	mmMale_key	45
4	Mouse female	mmFemale_key	47
5	cell	cell_key[["cell"]]	24
6	anolis_carolinensis	other_key[["anolis_carolinensis"]]	4
7	arabidopsis_thaliana	other_key[["arabidopsis_thaliana"]]	4
8	bos_taurus	other_key[["bos_taurus"]]	10
9	brachypodium_distachyon.flower_parts	other_key[["brachypodium_distachyon.flower_parts"]]	5
10	brachypodium_distachyon.whole_plant	other_key[["brachypodium_distachyon.whole_plant"]]	3
11	gallus_gallus	other_key[["gallus_gallus"]]	8
12	hordeum_vulgare.flower_parts	other_key[["hordeum_vulgare.flower_parts"]]	6
13	hordeum_vulgare.whole_plant	other_key[["hordeum_vulgare.whole_plant"]]	5
14	macaca_mulatta	other_key[["macaca_mulatta"]]	6
15	monodelphis_domestica	other_key[["monodelphis_domestica"]]	6
16	oryza_sativa.flower_parts	other_key[["oryza_sativa.flower_parts"]]	5
17	oryza_sativa.whole_plant	other_key[["oryza_sativa.whole_plant"]]	5
18	papio_anubis	other_key[["papio_anubis"]]	15
19	rattus_norvegicus	other_key[["rattus_norvegicus"]]	10
20	solanum_lycopersicum.flower_parts	other_key[["solanum_lycopersicum.flower_parts"]]	7
21	solanum_lycopersicum.whole_plant	other_key[["solanum_lycopersicum.whole_plant"]]	5
22	sorghum_bicolor.flower_parts	other_key[["sorghum_bicolor.flower_parts"]]	7
23	sorghum_bicolor.whole_plant	other_key[["sorghum_bicolor.whole_plant"]]	7
24	tetraodon_nigroviridis	other_key[["tetraodon_nigroviridis"]]	4
25	triticum_aestivum.flower_parts	other_key[["triticum_aestivum.flower_parts"]]	11
26	triticum_aestivum.whole_plant	other_key["triticum_aestivum.whole_plant"]]	4
27	xenopus_tropicalis	other_key[["xenopus_tropicalis"]]	5
28	zea_mays.flower_parts	other_key[["zea_mays.flower_parts"]]	9
29	zea_mays.whole_plant	other_key[["zea_mays.whole_plant"]]	7

These data frames have already specified colour, type, and an assigned random number to facilitate the start of plotting.

head(hgFemale_key)
            organ  colour      type     value
1        pancreas  orange digestion 10.373146
2           liver  orange digestion 19.723172
3           colon  orange digestion 14.853335
4     bone_marrow #41ab5d     other 19.681587
5 urinary_bladder  orange digestion 14.914273
6         stomach  orange digestion  2.667599

**Figure 2.**
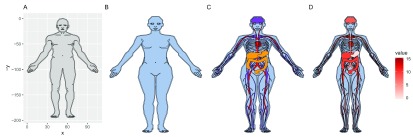
( **A**) Default plot generated by calling gganatogram(), ( **B**) adding female, plotting specified organs by ( **C**) colour, ( **D**) value.

The main function is called gganatogram(). By default, and without any arguments, it plots the outline of a male human with standard ggplot2 parameters. By adding just a few options, it is possible to quickly change to female, fill specified organs by selected colour, or fill the organs based on a value ( Figure 2).

library(gganatogram)
library(gridExtra)
organPlot <- data.frame(organ = c("heart", "leukocyte", "nerve", "brain",
"liver", "stomach", "colon"),
type = c("circulation", "circulation",  "nervous␣system", "nervous␣system",
"digestion", "digestion", "digestion"),
colour = c("red", "red", "purple", "purple", "orange", "orange", "orange"),
value = c(10, 5, 15, 8, 10, 0, 10),
stringsAsFactors=F)

A <- gganatogram() + ggtitle("A")
B <- gganatogram(fillOutline="#a6bddb", sex = "female") + theme_void() + 
ggtitle("B")
C <- gganatogram(data=organPlot, fillOutline="#a6bddb", organism="human",
sex="female", fill="colour")+ theme_void() + ggtitle("C")
D <- gganatogram(data=organPlot, fillOutline="#a6bddb", organism="human",
sex="female", fill="value") + theme_void() +
scale_fill_distiller(palette = "Reds", direction=1) + ggtitle("D")

grid.arrange(A, B, C, D, ncol=4)

## Use cases

This section provides additional plotting examples.

**Figure 3.**
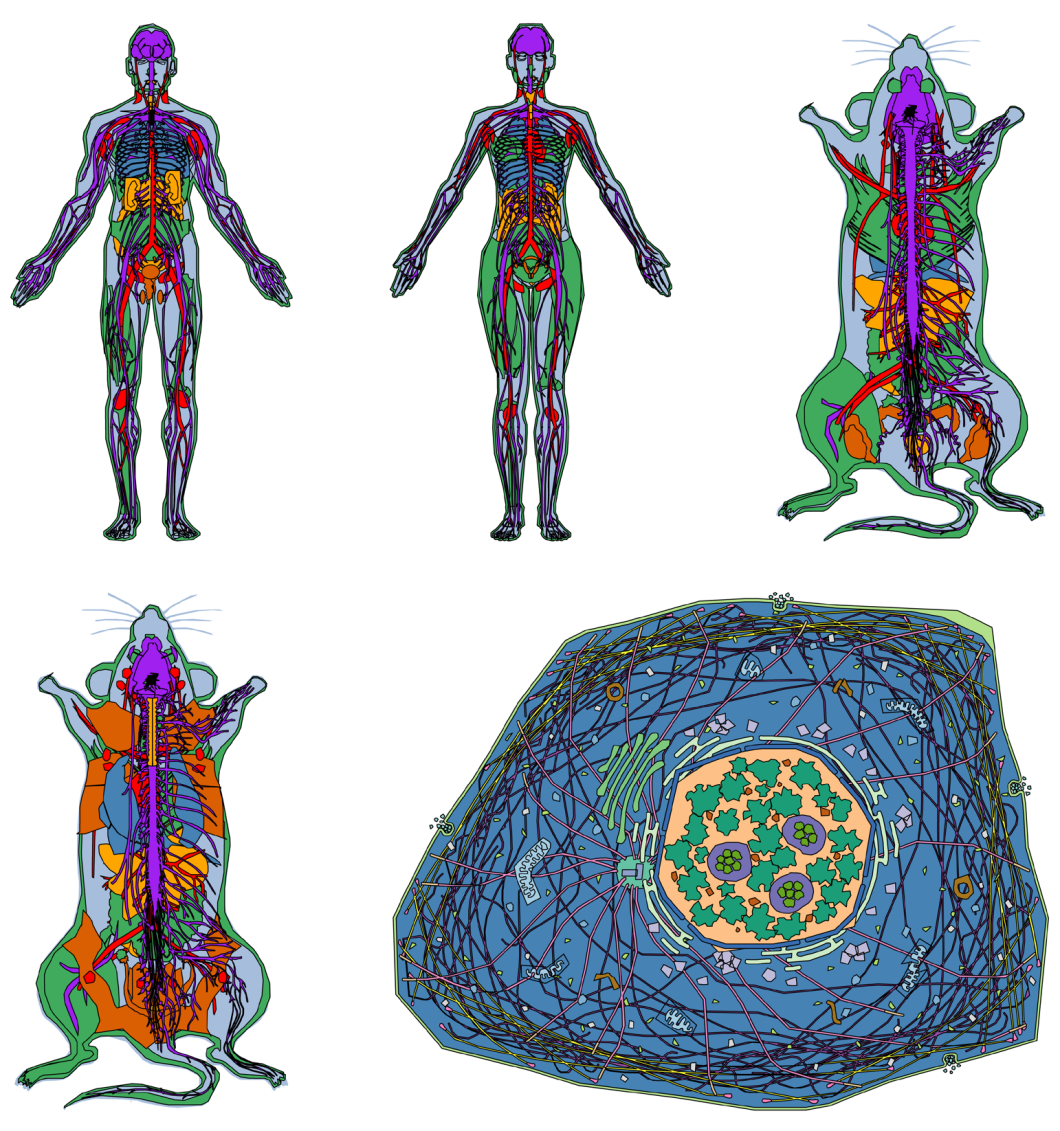
Displaying all tissues available for human and mouse, male and female. The colours are specified in the provided key data frames.

To plot all tissues per organism, use the provided key files that exist per organism and sex. This displays all tissues in the order of each data frame. To change the order in which organs are layered on top of each other, reorder the data frame to have those tissues at the bottom ( Figure 3).

library(gganatogram)
library(gridExtra)
hgMale <- gganatogram(data=hgMale_key, fillOutline="#a6bddb", organism="human",
 sex="male", fill="colour") + theme_void()+ coord_fixed()
hgFemale <- gganatogram(data=hgFemale_key, fillOutline="#a6bddb",
 organism="human", sex="female", fill="colour") + theme_void()+ coord_fixed()
mmMale <- gganatogram(data=mmMale_key, fillOutline="#a6bddb", organism="mouse",
 sex="male", fill="colour") + theme_void()+ coord_fixed()
mmFemale <- gganatogram(data=mmFemale_key, outline = T, fillOutline="#a6bddb",
 organism="mouse", sex="female", fill="colour") + theme_void()+ coord_fixed()
cell <- gganatogram(data=cell_key[["cell"]], outline = T, fillOutline="#a6bddb",
 organism="cell", fill="colour") + theme_void()+ coord_fixed() 
 
lay <- rbind(c(1,2,3), c(4,5, 5))
grid.arrange(hgMale, hgFemale, mmMale, mmFemale, cell, layout_matrix=lay)

To compare anatograms, e.g. draw one specific anatogram side by side and compare values, a long table has to be created with the type column changed to the variables to compare. The following code recreates ( Figure 1c).

normal <- data.frame(organ = c("heart", "leukocyte", "nerve", "brain", "liver", 
"stomach", "colon"),
value = c(10, 5, 1, 2, 2, 5, 5), 
type = rep(’Normal’, 7),
stringsAsFactors=F)

cancer <- data.frame(organ = c("heart", "leukocyte", "nerve", "brain", "liver", 
"stomach", "colon"),
value = c(5, 5, 10, 12, 15, 5, 10),  type = rep("Cancer", 7),
stringsAsFactors=F)

compareGroups <- rbind(normal, cancer)

gganatogram(data=compareGroups, fillOutline="white", organism="human",
sex="male", fill="value") +
              theme_void() +
              facet_wrap(~type) +
              scale_fill_distiller(palette = "Reds", direction=1)

**Figure 4.**
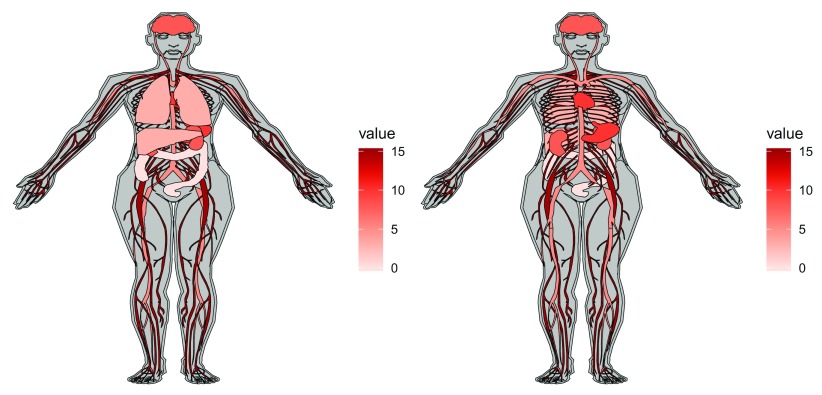
Changing the order of the data frame results in change in the layer of organs to plot.

To change the order of how organs are layered on top of each other, change the order of the data frame. The organs to plot on the top layer should be on the end of the data frame ( Figure 4).

organPlot <- data.frame(organ = c("heart", "leukocyte", "nerve", "brain",
"stomach", "colon", "lung", "kidney", "liver"),
value = c(10, 5, 15, 8, 10, 0, 3, 8, 3),
stringsAsFactors=F)

A <- gganatogram(data=organPlot, fillOutline="grey", organism="human",
sex="female", fill="value") + theme_void() +
scale_fill_distiller(palette = "Reds", direction=1)

organReorder <- c("lung", "liver", "colon", "nerve", "leukocyte",
"kidney",  "stomach", "brain", "heart")
organPlotReorder <- organPlot[match(organReorder, organPlot$organ),]
B <- gganatogram(data=organPlotReorder, fillOutline="grey",
organism="human", sex="female", fill="value") + theme_void() +
scale_fill_distiller(palette = "Reds", direction=1)

grid.arrange(A, B, ncol=2)

**Figure 5.**
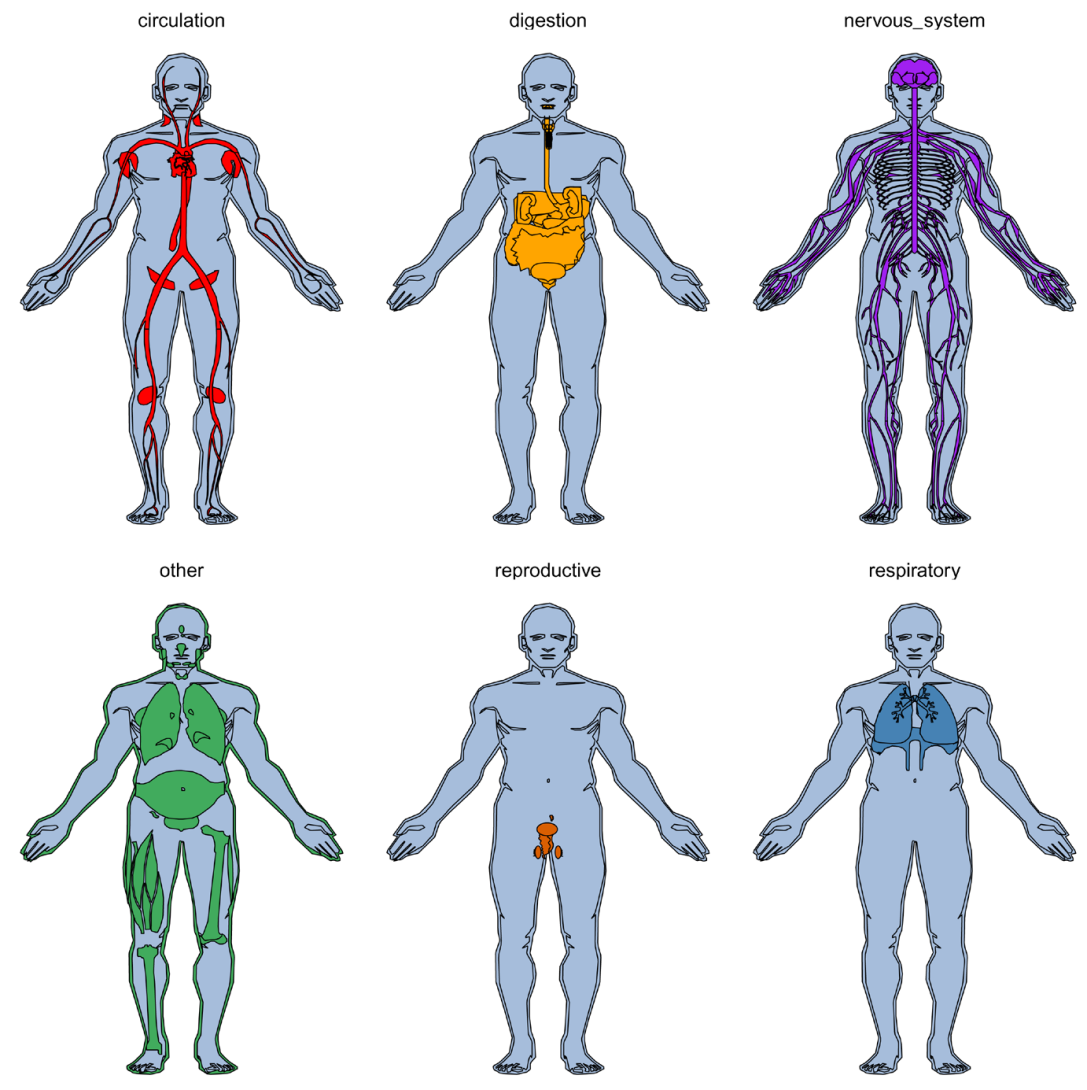
Faceting tissues based on type and displaying the corresponding colour.

Organs can also be separated by faceting, as per standard ggplot2 using facet_wrap ( Figure 5). This can help to display organs that are nested on top of each other. On the left, lungs and liver hides other plotted organs. This is corrected to the right in order to show the organs of interest.

library(gganatogram)

gganatogram(hgMale_key, fillOutline="#a6bddb", organism="human",
sex="male", fill="colour") +
theme_void() +
facet_wrap(~type)

**Figure 6.**
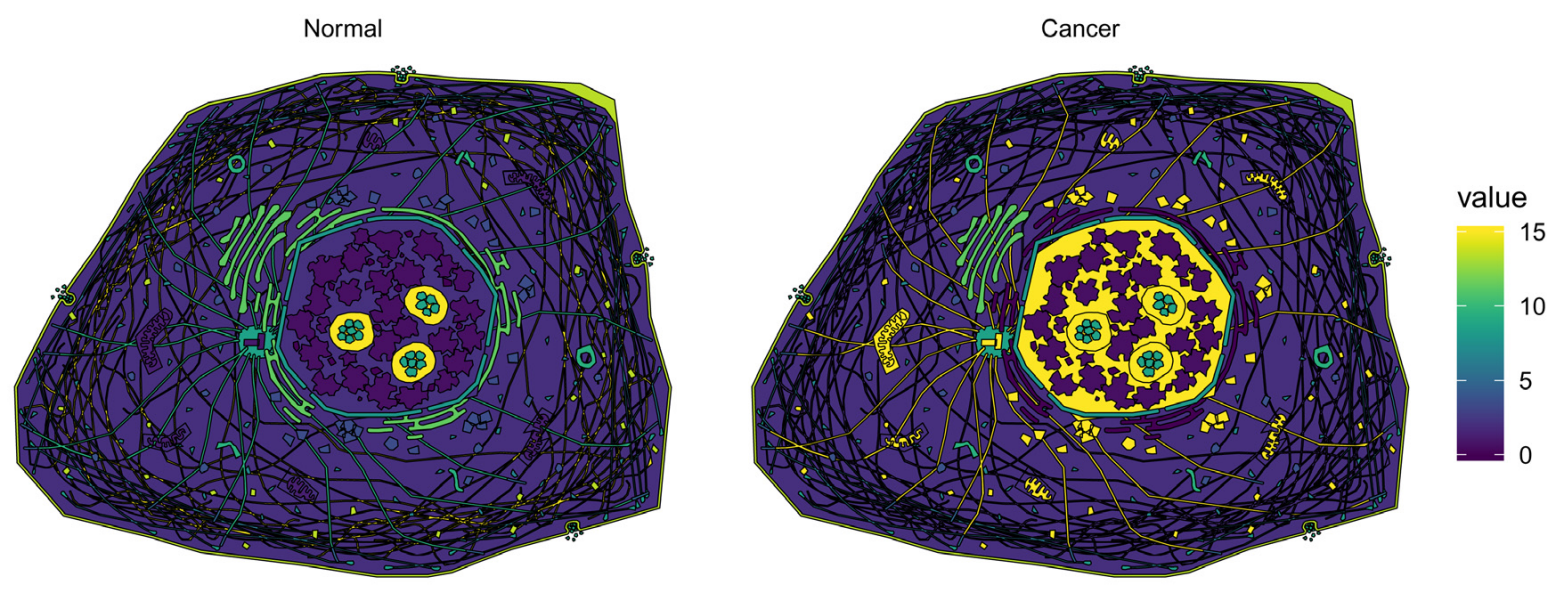
Cell diagram to plot values at specific cellular sub-locations.

The cell diagram will be useful for users to plot cellular sub-locations of proteins, mRNAs, or other molecules( Figure 6).

library(viridis)
library(dplyr)
normal <- cell_key[["cell"]]
normal$type <- "Normal"
cancer <- cell_key[["cell"]]
cellCompartments <- c("intermediate_filaments", "endoplasmic_reticulum", 
"centrosome", "microtubules", "nucleoplasm", "mitochondria", "endosomes", 
"lipid_droplets")
cancer[match(cellCompartments, cancer$organ),]$value <- c(2,0, rep(15, 6))
cancer$type <- "Cancer"
plotCell <- rbind(normal,cancer)
plotCell %>%
mutate(type = factor(type, levels=c("Normal", "Cancer")))%>%
gganatogram( outline = F, organism="cell", fill="value") + theme_void() +
coord_fixed() +  scale_fill_viridis() +facet_wrap(~type)

**Figure 7.**
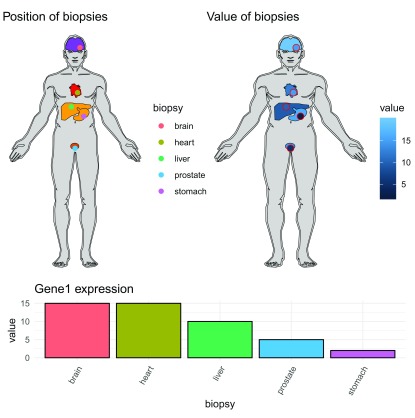
Geom points added to a gganatogram to show the location of tissue biopsies (top left) along with a barplot of biopsy expression for an example gene (bottom). Another option is to fill both tissues and points by value (top right). Red colour around plot added for emphasis.

Because I elected to use ggplot2 ^5^ for the package, the user can add additional layers from standard plots. This can be useful to show highlight features, such as metastasis, location of tissue biopsies, or gene expression of specific biopsies ( Figure 7).

library(gganatogram)
library(dplyr)
library(gridExtra)

biopsies <- data.frame(biopsy = c("liver", "heart", "prostate", "stomach", "brain"),
 x = c(50, 55, 53, 60, 57),
 y = c(60, 48, 95, 68, 10),
 value = c(10, 15, 5, 2, 15))
p <- hgMale_key %>%
 dplyr::filter(organ %in% c("liver", "heart", "prostate", "stomach", 
 "brain")) %>%
 gganatogram(fillOutline="lightgray", organism="human", sex="male",
 fill="colour") + theme_void() +
 ggtitle("Position␣of␣biopsies")
        
p <- p + geom_point(data = biopsies, pch=21, size=2, aes(x =x, y = -y,
 fill = biopsy, colour= biopsy))
p2 <- ggplot(biopsies, aes(x = biopsy, y = value, fill = biopsy)) +
 geom_bar(stat= "identity", col="black") +
 theme_minimal() +
 theme(legend.position= "none")+
 theme(axis.text.x = element_text(angle = 60, hjust = 1))+
 ggtitle("Gene1␣expression")
        
p3 <- hgMale_key%>%
 dplyr::filter(organ %in% c("liver", "heart", "prostate", "stomach", 
 "brain"))%>%
 gganatogram(fillOutline="lightgray", organism="human", sex="male", 
 fill="value") + theme_void() +
 ggtitle("Value␣of␣biopsies")+
 geom_point(data = biopsies, pch=21, size=3, aes(x =x, y = -y,
 fill = value), colour="red")
        
lay <- rbind(c(1,2), c(1,2), c(3, NULL))
grid.arrange(p, p3, p2, layout_matrix = lay)

**Figure 8.**
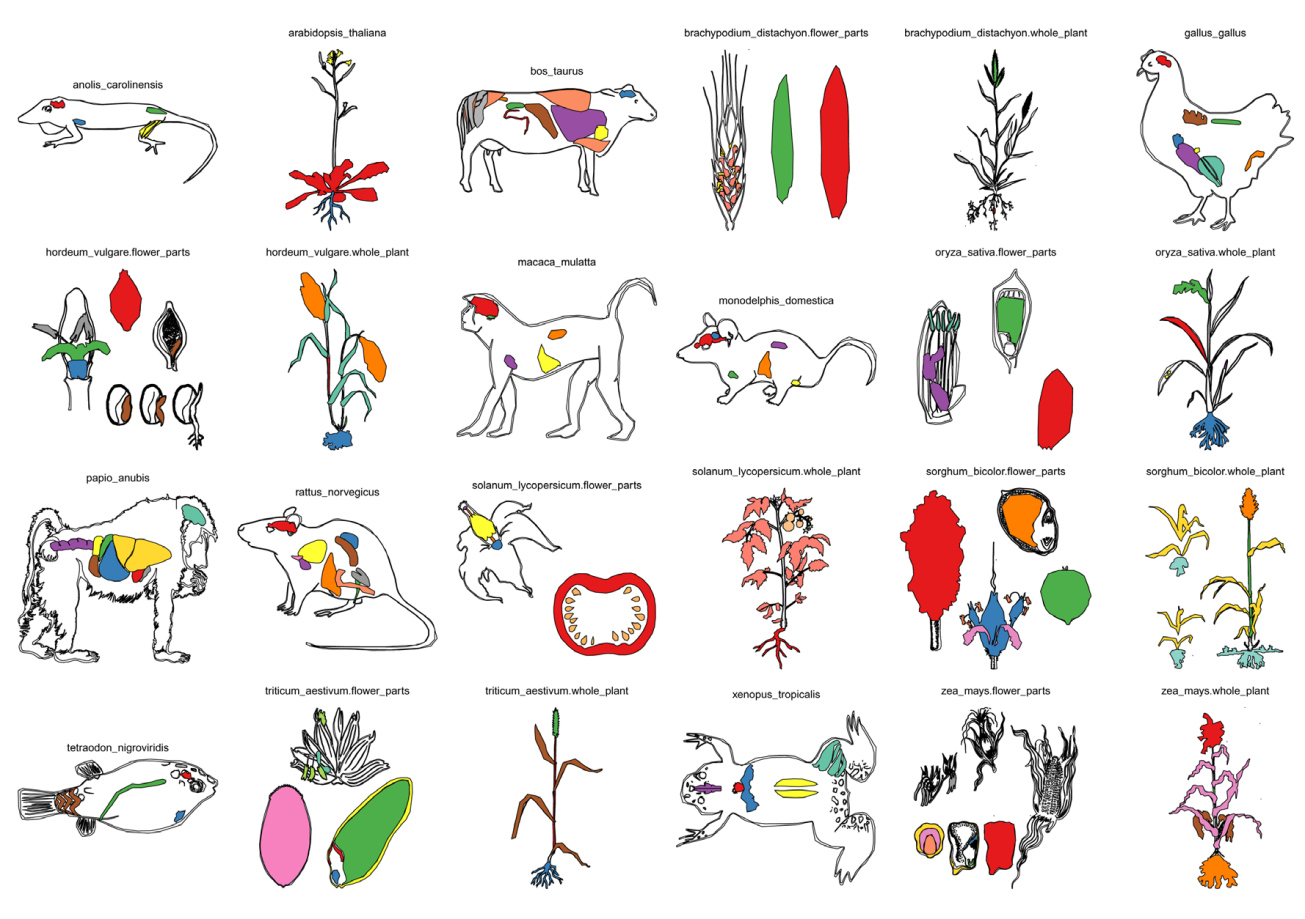
All 24 other organisms present in gganatogram with all their tissues plotted.

Other than human, mouse, and the cell diagram, gganatogram consists of 24 other organisms which can be called with the other_key ( Table 1). These consists of a mix of animals and plants. Unfortunately, these organisms are not as detailed which is apparent from the lower number of features to plot ( Table 1, Figure 8).

library(gridExtra)
plotList <- list()
for (organism in names(other_key)) {
    plotList[[organism]] <- gganatogram(data=other_key[[organism]], outline = T,
fillOutline="white", organism=organism, fill="colour")  +
               theme_void() +
               ggtitle(organism)+ 
               theme(plot.title = element_text(hjust=0.5, size=9)) + 
               coord_fixed()
}

do.call(grid.arrange,  c(plotList, ncol=6))

Furthermore, gganantogram has an online shiny app which can be used without any R installation. This app let people can select organisms, colour palette, select tissues and adjust values for the tissues. This should allow for researchers without any experience in R to be able to use gganatogram. The online shiny app is located at https://jespermaag.shinyapps.io/gganatogram/.

For R users, the app can easily be run locally with the following command:

library(shiny)
runGitHub("gganatogram", "jespermaag", subdir = "shiny")

This command checks for all required packages and installs them if needed.

## Summary

In summary, I have designed and implemented an R package to easily visualise anatograms based on ggplot2 ^5^ and the anatograms from Expression Atlas ^2^, which when combined create a powerful tool to plot and display tissue information.

The one line command to generate these plots should allow for users with even limited R knowledge to create informative anatograms for publications or presentations.

## Software availability

1. Link to version control repository containing the source code:
http://neuroconductor.org:8080/package/gganatogram
2. Link to development version:
github/jespermaag/gganatogram
3. Link to archived source code as at time of revision:
https://zenodo.org/record/1477474 ^9^
  Software license: **GPL-2**
