# Supplementary material for: gganatogram: An R package for modular visualisation of anatograms and tissues based on ggplot2
Source: F1000Res. 2018 Nov 20;7:1576. Originally published 2018 Sep 28. [Version 2] doi: 10.12688/f1000research.16409.2 (PMC6208569; doi:10.12688/f1000research.16409.2)
Supplement: Peer review history

# Peer review: Referee response for version 2

**DOI:** 10.5256/f1000research.18533.r40885

**Published:** 2018-11-21

**Authors:** Saskia Freytag

**Affiliations:** 1 Population Health and Immunity Division, Walter and Eliza Hall Institute (WEHI), Department of Medical Biology, University of Melbourne, Parkville, Vic, Australia

Really nice update to the article!

I have read this submission. I believe that I have an appropriate level of expertise to confirm that it is of an acceptable scientific standard.

# Peer review: Referee response for version 1

**DOI:** 10.5256/f1000research.17925.r39943

**Published:** 2018-10-30

**Authors:** Saskia Freytag

**Affiliations:** 1 Population Health and Immunity Division, Walter and Eliza Hall Institute (WEHI), Department of Medical Biology, University of Melbourne, Parkville, Vic, Australia

This article describes a new R package, which allows easy plotting of discrete and continuous measurements onto human and mouse anatomy.

 This is a really valuable R package contribution, as it fills a real void in the current infrastructure. I found the code examples in the manuscript intuitive and easy to run. It is great that the author adopts the popular ggplot2 grammar as well as a tidy data structures.

 Minor comments:

1. It would be useful to know how many tissues (and which) can be plotted using this package.
2. An example of changing the order of the data frame to change the layering should be added.
3. I encountered the following error when trying to install the package through neuroconductor:
  Error in latest_neuroc_release(release = "stable") :
  unused argument (release = "stable")

Thank you for making all code (even for processing) publicly available.

I have read this submission. I believe that I have an appropriate level of expertise to confirm that it is of an acceptable scientific standard.

# Peer review: Referee response for version 1

**DOI:** 10.5256/f1000research.17925.r39255

**Published:** 2018-10-22

**Authors:** Helder I. Nakaya

**Affiliations:** 1 University of São Paulo, São Paulo, Brazil

The author describes an R package that displays discrete and continuous data onto anatomical structures. The structures are based on mouse and human anatograms from the Expression Atlas project and the grammar from ggplot2 R library. The code example was easy to run and the necessary input data was intuitive and simple. 

 The author can increase its usage by providing a webtool (even one based on shiny) that takes as input a csv or tsv table with predefined columns. This would allow physicians and scientists with no background in bioinformatics to easily display their data. 

 Also, it would be useful to create an anatogram for the human brain and one for the different compartments of a cell (nucleus, mitochondria, etc).

I have read this submission. I believe that I have an appropriate level of expertise to confirm that it is of an acceptable scientific standard.
